# Supplementary material for: Barriers and motivators of contraceptive use among young people in Sub-Saharan Africa: A systematic review of qualitative studies
Source: PLoS One. 2021 Jun 4;16(6):e0252745. doi: 10.1371/journal.pone.0252745 (PMC8177623; doi:10.1371/journal.pone.0252745)
Supplement: S2 File — (DOCX) [file pone.0252745.s002.docx]

1. Patient DR, Orr NM. What teenagers and young adults have to say about condoms and using condoms. AIDS analysis Africa. 2000;10(6):11.
2. Otoide VO, Oronsaye F, Okonofua FE. Why Nigerian adolescents seek abortion rather than contraception: evidence from focus-group discussions. International family planning perspectives. 2001:77-81.
3. Castle S. Factors influencing young Malians' reluctance to use hormonal contraceptives. Studies in family planning. 2003;34(3):186-99.
4. Flaherty A, Kipp W, Mehangye I. ‘We want someone with a face of welcome’: Ugandan adolescents articulate their family planning needs and priorities. Tropical doctor. 2005 Jan;35(1):4-7.
5. Tabane NS, Peu MD. Perceptions of female teenagers in the Tshwane District on the use of contraceptives in South Africa. curationis. 2015;38(2):1-7.
6. Ochako R, Mbondo M, Aloo S, Kaimenyi S, Thompson R, Temmerman M, Kays M. Barriers to modern contraceptive methods uptake among young women in Kenya: a qualitative study. BMC public health. 2015;15(1):118.
7. Hall K, Manu A, Morhe E, Zochowski M, Boakye A, Challa S, Loll D, Dozier J, Harris L, Adanu R, Dalton VK. Understanding “Bad Girl:” qualitative findings on stigma and adolescent sexual and reproductive health in Ghana. Contraception. 2015;92(4):393-4.
8. Hokororo A, Kihunrwa AF, Kalluvya S, Changalucha J, Fitzgerald DW, Downs JA. Barriers to access reproductive health care for pregnant adolescent girls: a qualitative study in Tanzania. Actapaediatrica. 2015 Dec;104(12):1291-7.
9. Capurchande R, Coene G, Schockaert I, Macia M, Meulemans H. “It is challenging… oh, nobody likes it!”: a qualitative study exploring Mozambican adolescents and young adults’ experiences with contraception. BMC women's health. 2016 Dec;16(1):48.
10. Hall KS, Manu A, Morhe E, Challa S, Loll D, Dozier J, Zochowski M, Boakye A, Harris L, Adanu R, Dalton V. Stigma and the social environment of adolescent sexual and reproductive health in ghana: understanding “bad girl”. Journal of Adolescent Health. 2016 Feb 1;58(2):S4.
11. Burke E, Kébé F, Flink I, van Reeuwijk M, le May A. A qualitative study to explore the barriers and enablers for young people with disabilities to access sexual and reproductive health services in Senegal. Reproductive health matters. 2017 Jun 12;25(50):43-54.
12. Hall KS, Manu A, Morhe E, Dalton VK, Challa S, Loll D, et al. Bad girl and unmet family planning need among Sub-Saharan African adolescents: the role of sexual and reproductive health stigma. Qualitative research in medicine & healthcare. 2018 May 30;2(1):55.
13. Rokicki S, Merten S. The context of emergency contraception use among young unmarried women in Accra, Ghana: a qualitative study. Reproductive health. 2018 Dec;15(1):1-0.
