# Supplementary material for: Barriers and motivators of contraceptive use among young people in Sub-Saharan Africa: A systematic review of qualitative studies
Source: PLoS One. 2021 Jun 4;16(6):e0252745. doi: 10.1371/journal.pone.0252745 (PMC8177623; doi:10.1371/journal.pone.0252745)
Supplement: Attachment — Submitted filename: PONE-D-20-10773_reviewer.pdf [file pone.0252745.s003.pdf]

PLOS ONE

Barriers to and motivations for contraceptive use among adolescents and young women in Sub-Saharan Africa: A systematic review of qualitative studies

--Manuscript Draft--

**Manuscript Number:**	PONE-D-20-10773
**Article Type:**	Research Article
**Full Title:**	Barriers to and motivations for contraceptive use among adolescents and young women in Sub-Saharan Africa: A systematic review of qualitative studies
**Short Title:**	Contraceptive uptake among adolescents and young women in Sub-Saharan Africa.
**Corresponding Author:**	Luchuo Engelbert Bain, MD, MPH, MSc, PhDc<br>Centre for Population Studies and Health Promotion, Cameroon, and Athena Institute, Faculty of Earth and Life Sciences, VU Amsterdam, Netherlands.<br>Yaounde, CAMEROON
**Keywords:**	contraception, adolescents, Sub - Saharan Africa, young women, qualitative studies, barriers, motivations
**Abstract:**	Background: In sub-Saharan Africa, about 80% of young women either use a traditional method or do not use any form of contraception at all. The aim of this review was to ascertain the barriers and motivations for the uptake of contraceptives among adolescents and young women in Sub–Saharan Africa.<br>Materials and Methods: An Electronic literature search was conducted in PubMed, EMBASE, Ebsco/PsycINFO and Scopus. A total of 4,457 publications were identified and 2626 initially screened based on Preferred Reporting Items for Systematic Reviews and Meta-Analyses (PRISMA). A total of 13 qualitative studies were retained for the final analysis based on the Joanna Briggs criteria for assessing qualitative studies. The systematic review is registered on PROSPERO with identifier CRD42018081877.<br>Results: Supportive social support networks, respect for privacy and confidentiality, readily available, affordable and accessible contraceptives, as well as the desire to prevent an unintended pregnancy and sexually transmitted infections improved the uptake of contraceptive use among adolescents and young women in sub-Saharan Africa. Despite these motivations, myriad of personal, societal, and health systems-based barriers including myths and misconceptions, known side effects of contraceptives, prohibitive social norms, and negative attitude of health professionals were major barriers to contraceptive use among women in this age group.<br>Conclusion: Sub-Saharan African countries may not be able to achieve the Sustainable Development Goal 3.8 target of health for all by the year 2030 if the barriers to contraceptive use persist, especially among adolescents and young women. Interventions to improve uptake are to be inter sectoral and multi-layered, in order to carefully address the personal, cultural, organizational and political dimensions of contraceptive use.
**Order of Authors:**	Luchuo Engelbert Bain, MD, MPH, MSc, PhDc
	Amu Hubert
	Tarkang Elvis Enowbeyang
**Additional Information:**	
**Question**	**Response**
**Financial Disclosure**<br><br>Enter a financial disclosure statement that describes the sources of funding for the work included in this submission. Review the submission guidelines for detailed requirements. View published research	NO : The funders had no role in study design, data collection and analysis, decision to publish, or preparation of the manuscript

Powered by Editorial Manager® and ProduXion Manager® from Aries Systems Corporation

articles from *PLOS ONE* for specific examples.<br><br>This statement is required for submission and **will appear in the published article** if the submission is accepted. Please make sure it is accurate.<br><br>**Unfunded studies**<br>Enter: *The author(s) received no specific funding for this work.*<br><br>**Funded studies**<br>Enter a statement with the following details:<br>• Initials of the authors who received each award<br>• Grant numbers awarded to each author<br>• The full name of each funder<br>• URL of each funder website<br>• Did the sponsors or funders play any role in the study design, data collection and analysis, decision to publish, or preparation of the manuscript?<br>• **NO** - Include this sentence at the end of your statement: *The funders had no role in study design, data collection and analysis, decision to publish, or preparation of the manuscript.*<br>• **YES** - Specify the role(s) played.<br><br>* typeset	
**Competing Interests**<br><br>Use the instructions below to enter a competing interest statement for this submission. On behalf of all authors, disclose any competing interests that could be perceived to bias this work—acknowledging all financial support and any other relevant financial or non-financial competing interests.<br><br>This statement **will appear in the published article** if the submission is accepted. Please make sure it is accurate. View published research articles from *PLOS ONE* for specific examples.	The authors have declared that no competing interests exist.

**NO authors have competing interests**<br><br>Enter: *The authors have declared that no competing interests exist.*<br><br>**Authors with competing interests**<br><br>Enter competing interest details beginning with this statement:<br><br>*I have read the journal's policy and the authors of this manuscript have the following competing interests: [insert competing interests here]*<br><br>* typeset	
**Ethics Statement**<br><br>Enter an ethics statement for this submission. This statement is required if the study involved:<br><br>• Human participants<br>• Human specimens or tissue<br>• Vertebrate animals or cephalopods<br>• Vertebrate embryos or tissues<br>• Field research<br><br>Write "N/A" if the submission does not require an ethics statement.<br><br>General guidance is provided below. Consult the submission guidelines for detailed instructions. **Make sure that all information entered here is included in the Methods section of the manuscript.**	N/A

**Format for specific study types**<br><br>**Human Subject Research (involving human participants and/or tissue)**<br>• Give the name of the institutional review board or ethics committee that approved the study<br>• Include the approval number and/or a statement indicating approval of this research<br>• Indicate the form of consent obtained (written/oral) or the reason that consent was not obtained (e.g. the data were analyzed anonymously)<br><br>**Animal Research (involving vertebrate animals, embryos or tissues)**<br>• Provide the name of the Institutional Animal Care and Use Committee (IACUC) or other relevant ethics board that reviewed the study protocol, and indicate whether they approved this research or granted a formal waiver of ethical approval<br>• Include an approval number if one was obtained<br>• If the study involved *non-human primates*, add *additional details* about animal welfare and steps taken to ameliorate suffering<br>• If anesthesia, euthanasia, or any kind of animal sacrifice is part of the study, include briefly which substances and/or methods were applied<br><br>**Field Research**<br><br>Include the following details if this study involves the collection of plant, animal, or other materials from a natural setting:<br>• Field permit number<br>• Name of the institution or relevant body that granted permission	
**Data Availability**<br><br>Authors are required to make all data underlying the findings described fully available, without restriction, and from the time of publication. PLOS allows rare exceptions to address legal and ethical concerns. See the PLOS Data Policy and FAQ for detailed information.	Yes - all data are fully available without restriction

A Data Availability Statement describing where the data can be found is required at submission. Your answers to this question constitute the Data Availability Statement and **will be published in the article**, if accepted.<br><br>**Important:** Stating 'data available on request from the author' is not sufficient. If your data are only available upon request, select 'No' for the first question and explain your exceptional situation in the text box.<br><br>Do the authors confirm that all data underlying the findings described in their manuscript are fully available without restriction?	
**Describe where the data may be found in full sentences. If you are copying our sample text, replace any instances of XXX with the appropriate details.**<br><br>• If the data are **held or will be held in a public repository**, include URLs, accession numbers or DOIs. If this information will only be available after acceptance, indicate this by ticking the box below. For example: *All XXX files are available from the XXX database (accession number(s) XXX, XXX.).*<br>• If the data are all contained **within the manuscript and/or Supporting Information files**, enter the following: *All relevant data are within the manuscript and its Supporting Information files.*<br>• If neither of these applies but you are able to provide **details of access elsewhere**, with or without limitations, please do so. For example:<br><br>*Data cannot be shared publicly because of [XXX]. Data are available from the XXX Institutional Data Access / Ethics Committee (contact via XXX) for researchers who meet the criteria for access to confidential data.*<br><br>*The data underlying the results presented in the study are available from (include the name of the third party*	All selected articles can be consulted and explored in case of need.

and contact information or URL).<br>• This text is appropriate if the data are owned by a third party and authors do not have permission to share the data.<br><br>* typeset	
Additional data availability information:	Tick here if the URLs/accession numbers/DOIs will be available only after acceptance of the manuscript for publication so that we can ensure their inclusion before publication.

Powered by Editorial Manager® and ProduXion Manager® from Aries Systems Corporation

Manuscript

Click here to access/download;Manuscript;Systematic Review - adolescent contraception ([plosone]).docx

Barriers to and motivations for contraceptive use among adolescents and young women in Sub-Saharan Africa: A systematic review of qualitative studies

Luchuo Engelbert Bain[1,2] Hubert Amu[3], Elvis Enowbeyang Tarkang[3]

1. Athena Institute, Faculty of Science, Vrije Universiteit, Amsterdam, The Netherlands
2. Global South Health Research and Services, GSHS, The Netherlands.
3. Department of Population and Behavioural Sciences, School of Public Health, University of Health and Allied Sciences, Hohoe, Ghana

Corresponding author:

Luchuo Engelbert Bain

Email address: lebaiins@gmail.com

Postal Address: Athena Institute, Faculty of Science, Vrije Universiteit, Amsterdam.

de Boelelaan 1085, 1081 HV Amsterdam.

Abstract

Background: In sub-Saharan Africa, about 80% of young women either use a traditional method or do not use any form of contraception at all. The aim of this review was to ascertain the barriers and motivations for the uptake of contraceptives among adolescents and young women in Sub–Saharan Africa.

Materials and Methods: An Electronic literature search was conducted in PubMed, EMBASE, Ebsco/PsycINFO and Scopus. A total of 4,457 publications were identified and 2626 initially screened based on Preferred Reporting Items for Systematic Reviews and Meta-Analyses (PRISMA). A total of 13 qualitative studies were retained for the final analysis based on the Joanna Briggs criteria for assessing qualitative studies. The systematic review is registered on PROSPERO with identifier CRD42018081877.

Results: Supportive social support networks, respect for privacy and confidentiality, readily available, affordable and accessible contraceptives, as well as the desire to prevent an unintended pregnancy and sexually transmitted infections improved the uptake of contraceptive use among adolescents and young women in sub-Saharan Africa. Despite these motivations, myriad of personal, societal, and health systems-based barriers including myths and misconceptions, known side effects of contraceptives, prohibitive social norms, and negative attitude of health professionals were major barriers to contraceptive use among women in this age group.

Conclusion: Sub-Saharan African countries may not be able to achieve the Sustainable Development Goal 3.8 target of health for all by the year 2030 if the barriers to contraceptive use persist, especially among adolescents and young women. Interventions to improve uptake are to be inter sectoral and multi-layered, in order to carefully address the personal, cultural, organizational and political dimensions of contraceptive use.

Keywords: Sub-Saharan Africa, Contraceptive Use, Young People, Barriers, Motivation.

Introduction

Pregnancy and childbirth among adolescents and young women remain a major public health concern worldwide [1]. In low- and middle-income countries (LMICs), approximately 16 million girls aged 15 to 19 years give birth every year, with 2.5 million of these girls under the age of 16 years [1]. In sub-Saharan Africa (SSA), 13% of pregnancies end up in abortions and 97% of these are classified as unsafe [2]. Optimal contraceptive use alone has the capacity of reducing the burden of unintended pregnancies and abortions by one third [3,4]. High levels of contraceptive failure or discontinuation are accrued predominantly to the use of traditional methods of contraception (coitus interruptus [withdrawal method], lactational amenorrhea method, calendar/rhythm method, cervical mucus method, and abstinence), and as a consequence increase the overall burden of unintended pregnancies, and abortions in Sub-Saharan Africa [4].

With many SSA countries still having challenges with providing optimal access to safe abortion care, non–use or inconsistent use of these effective contraceptive methods account for most of the unsafe abortions, and consequently high maternal mortality and morbidity rates, especially among young people [1].These challenges and their resultant morbidities and mortalities come at the backdrop that sub-Saharan African countries signed up to meeting the Sustainable Development Goal (SDG)3.8 target of achieving health for all by the year 2030 [5,6]. Achieving health for all implies that the countries have to, among other things increase the level of contraceptive use among their populace especially young people. Young people according to the World Health Organisation [7] are people 10–24 years old.

Intrauterine devices and contraceptive implants, also called Long-Acting Reversible Contraceptives (LARC) are the most effective reversible contraceptive methods [8] and are highly recommended for young people [9].Despite high levels of awareness that have been reported lately regarding contraceptives in SSA, utilisation has been overwhelmingly sup-optimal [11–16]. In sub-Saharan Africa, however, about 80% of young people use traditional methods, or do not use any form of contraception at all [8]. The aim of this review was, therefore, to ascertain the barriers and facilitators regarding the uptake of contraceptives among young people in Sub–Saharan Africa. Findings from this review could be potentially relevant in adequately planning and implementing public health interventions among this target group.

Methods

A comprehensive search was performed in the bibliographic databases: PubMed, Embase.com, Ebsco/PsycINFO and Scopus in collaboration with the medical librarian of the Vrije Universiteit, Amsterdam. A review protocol was developed based on the Preferred Reporting Items for Systematic Reviews and Meta-Analyses (PRISMA)-statement (www.prisma-statement.org). Databases were searched from inception up to 10th September, 2019. The following terms were used (including synonyms and closely related words) as index terms or free-text words: “Africa South of the Sahara”, “sub-Saharan Africa” “Adolescent”, “Young women”, “young people” “Family Planning” , “Contraception” , (contraceptives) “Birth Control”, “young female”, “teen”, “family planning”, “barrier”, “factor”, “enablers”, “facilitators”, “predictor”, “determinant”. The search was performed withou[illegible]te, language or publication status restriction. Duplicate articles were excluded. The full search strategies for all databases can be found in the Supplementary Information. The systematic review is registered on PROSPERO with identifier CRD42018081877.

Only qualitative studies were retained for the final review. To ensure trust-worthiness of the themes and sub-themes generated, two independent researchers were involved in the selection of articles. The reviewers met and decided upon the articles to be retained for final review after

discussing and coming to a consensus. In addition to these, two reviewers assessed clear reporting of aims and objectives of the study, adequate description of the context in which the research was carried out, adequate description of the sample and the methods by which the sample was identified and recruited, adequate description of the methods used to collect data, and adequate description of the methods used to analyse data. The Joanna Briggs criteria for assessing qualitative studies were used [17] with particular focus on the critical appraisal checklist for qualitative research, to determine the eligibility of studies included in the final review. We used conceptual thematic analysis to re-categorise the barriers and motivations reported by the studies into major themes, sub-themes, and their respective codes. These were then organised in the form of tables.

Results

Pursuant to the electronic search through the relevant databases, a total of 4,457 publications were found. Out of this, 2,626 articles were screened based on article title and abstracts. At this stage, 2,475 were excluded due to unfit title and abstract. The remaining 151 full text articles were further screened for eligibility and 134 were expunged. A total of 13 studies were finally retained. Figure 1 presents the PRISMA flow diagram on the literature selection.

The studies spanned the period 2000 to 2018 (See Table l). Four were conducted in Ghana and two in South Africa. Mali, Nigeria, Uganda, Kenya, Tanzania, Mozambique, and Senegal each recorded one study. While 12 of the studies generally targeted young people, one focused on young people with disabilities. Concerning methods of data collection, seven of the studies used

only in-depth interviews, three studies used only focus group discussions, and one adopted IDI and FGD. Of the remaining two studies, one added responses of young people in a general discussion to FGDs while the other one added informal conversation and observation to IDI and FGD.

For 12 of the studies, sample sizes used ranged from 15 to 149. Ten of the studies focused on all contraceptives while one focused on condoms. The remaining two also focused on emergency contraceptives and modern contraceptives respectively (See table 1).

Motivations for contraceptive use

Eight out of the 13 studies reported on motivations for contraceptive use. Table 2 presents the motivations for contraceptive use as reported by the eight studies. Five main themes were realised from the review. These were: social support, protection of identity, ready availability, affordability and access, effectiveness in preventing unintended pregnancy and Sexually Transmitted Infections (STIs), and other motivations. The "other" theme was labelled as such because the motivations under the theme were mentioned by just one study in each case.

Concerning social support, three sub-themes were realised. These were: support from friends and peers, support from family, and support from other people who are neither friends nor family. Two studies reported on friends being a major motivation for their utilization of contraceptives [23,29]. Burke et al. [29] and Hall et al. [30] also reported on family being important motivations for young people's contraceptive use. Tabane and Peu [23] also indicated that apart from friends, there were "significant others" who motivate young people do utilise contraceptives.

Two sub-themes were realised from the theme on protection of identity. These were anonymity and confidentiality. These two issues were made one theme because they both deal with

concealing the identity of the young people regarding their utilization of contraceptives. Two studies made findings on anonymity [19,29] where the study participants for instance, indicated that the use of purchase points which were mainly vending machines offered acceptable levels of anonymity and thus, motivated them to purchase and use the contraceptives. Confidentiality was also associated with patent medicine stores in 2 studies [20,29]. Lack of confidence in sellers of modern contraceptives was a major hindrance for young people's utilization of contraceptives [20,29].

Three studies also mentioned ready availability, affordability, and access to contraceptives as a major motivation for their utilization of contraceptives. Accessibility was for instance found by Patient and Orr [1] and Burke et al. [29] while availability and affordability were both found in Castle's [21] study. According to the young people in these studies, condoms for instance were cheap and this made it possible to afford and use them. The condoms were also readily available at various points of sale for easy purchase whenever needed.

The effectiveness of contraceptives in preventing unintended pregnancy and STIs was also reported as a motivation for their utilisation. Castle [21], Tabane and Peu [23], and Rokicki and Merten [31] all made findings on the effectiveness of contraceptives in preventing unintended pregnancy. Castle [21] also reported that a motivation for using condoms among young people was the fact that it made it possible for them to avoid contracting STIs. Other motivations mentioned by the studies comprised the need to maximise fertility in future (so as to gain status through childbearing) [21], service providers being of the same sex as the young people [22], and perceived quality of pharmacy shops [29].

Barriers to contraceptive use

Table 3 presents the barriers to contraceptive use which were reported by all the 13 studies included in our analyses. Three main themes were identified from the review. These were personal, societal, and health systems-based barriers. With personal barriers, five sub-themes were observed. They were: myths and misconceptions, lack/inadequate knowledge, negative attitude towards contraceptive use, known side effects of contraceptives, and financial challenges. Myths and misconceptions were the most reported personal barriers to contraceptive use. The sub-themes reported under myths and perceptions were lack of trust in contraceptives (especially condoms), colour and size of available condoms considered unsuitable, contraceptive use encourages promiscuity and straying (cheating), contraceptive use reduces sexual pleasure, risk of future infertility with contraceptive use, and perceived ineffectiveness of contraceptives in preventing conception. Five studies also reported the known side effects of contraceptives including weight gain, bleeding, high blood pressure, headache, and disruption of menstrual cycle as personal barriers to contraceptive use among the young people.

Societal-based barriers were reported by nine studies, comprising two sub-themes which were social consequences of contraceptive use and social norms. The specific societal based barriers to contraceptive use were: divorce, accusations of witchcraft, stigma, tag of being promiscuous, disproval of contraceptive use by friends and colleagues as well as family and the larger society, societal prohibition of discussions on issues concerning contraception, contraception being considered an issue only for females, and religious prohibitions.

Health systems-based barriers which were reported by 7 of the studies comprised five sub-themes; lack of privacy and confidentiality at health facilities, negative attitude of health professionals, long waiting time, poor communication between health professionals and young people, and physical inaccessibility of buildings by the persons with disability as the sub-themes.

The most reported sub-theme was negative attitude of health professionals which comprised being treated disrespectfully, being entirely refused contraceptive services, being ignored teaching about contraceptives, and discrimination.

Discussion

In this review, we explored barriers and motivations for contraceptive use among younger people in sub-Saharan Africa using 13 qualitative publications. The motivations were re-categorised into social support, protection of identity, ready availability, affordability and access, effectiveness in preventing unintended pregnancy and STIs, and other motivations. The barriers were also re-categorised into personal, societal, and health systems-based barriers.

Social support was realised in our review as an important motivation for contraceptive use. Tabane and Peu [23] for instance posited that approval from friends and positive peer influence from peers greatly encourage young people to utilise contraceptives. Burke et al. [29] and Hall et al. [30] also noted that family support is an instrumental protective factor for young people's utilisation of contraceptives. We also realised from our review that when anonymity and confidentially are assured and there is ready availability, affordability, and access, young people are highly motivated to patronise contraceptives and contraceptive services. This is an issue of importance because, young people in sub-Saharan Africa have been known to have grave unmet needs for contraceptive use which stems from their inability to access, afford, and/or be guaranteed confidentiality when they make efforts to utilise contraceptives [32,33].

Despite the motivations realised in our review, we observed that myriad of barriers also inhibit young people from utilising contraceptives and this has negative implications for sub-Saharan African countries towards attainment of health for all by the year 2030. Key among these challenges are myths and misconceptions. Young people according to Otoide et al. [20],

Hokororo et al. [26], Capurchande et al. [27], and Rokicki and Merten[31] for instance, erroneously perceived that contraceptives were ineffective in preventing conception. They also believed that contraceptive use posed risks of future infertility [20,21,22,24]. The myth and misconceptions realised in our review could have been due to lack of knowledge which Otoide et al. [20], Flaherty et al. [22], Tabane and Peu [23], Hokororo et al. [26], Capurchande et al. [27], Burke et al. [29] and Rokicki and Merten [31] found as comprising poor knowledge on the mechanism of action of contraceptives and on how to utilise them and lack of reliable, trusted and non-judgmental sources of information on contraceptives was also reported by seven of the studies. The findings of our review regarding knowledge points to its essential role in the reproductive health decision making of young people including contraception [34–37].

Our review also revealed that the known side effects of contraception such as: weight gain, bleeding, headaches, high blood pressure, and disruption of the menstrual cycle were personal barriers to contraceptive use among the young people. If such fears are not effectively managed through innovative approaches which reassure them of the important reasons for utilisation, such young people would continue to shy away from contraceptive use and this would not auger well for the achievement of universal health coverage by countries in sub-Saharan Africa. Besides, financial challenges which were mainly about the unaffordability of contraceptives and contraceptive services remained major barriers to the young people in their quest to utilise the contraceptives according to Burke et al. [29] and Rokicki and Merten [31]. This finding, however, contradicts arguments by Castle [21] that young people in her study felt contraceptives were affordable and this served as a motivation for them in utilizing such contraceptives.

Attitude of health professionals towards clients is an important determinant of health care utilisation [38–40]. Negative attitude of health professionals towards young people was reported

by Flaherty et al. [22], Tabane and Peu [23], Hokororo et al. [26], Burke et al. [29], and Hall et al. [30] as a health systems-based barrier to contraceptive use among young people and this manifested in being treated disrespectfully, being entirely refused contraceptive services, being ignored about contraceptives, and discrimination.

Social norms are powerful societal factors which predict the health seeking behaviour of people. In our review, we realised that these norms militated against the utilisation of contraceptives among young people in the form of disproval of contraceptive use by friends and colleagues, disproval of contraceptive use by family and larger society, societal prohibition of discussions on issues concerning contraception, and contraception being considered an issue only for females. The continuous prevalence of such inhibitive social norms in sub-Saharan Africa would only continue to preclude young people in these countries from utilizing contraceptives and thus making it impossible for the countries to achieve universal levels of contraceptive use.

Conclusions

Our review revealed social support, protection of identity, ready availability, affordability and access, effectiveness in preventing unintended pregnancy and STIs as motivations for young people in sub-Saharan Africa to use contraceptives. Despite these motivations, grave personal, societal, and health systems-based barriers prevail and prevent the young people from utilizing the contraceptives. These barriers include myths and misconceptions, lack/inadequate knowledge on contraceptives, known side effects of contraceptives, financial challenges, prohibitive social norms, negative attitude of health professionals, and poor communication between health professionals and young people. The implication of these barriers is that Sub-Saharan African countries may not be able to achieve the SDG target of health for all by the year 2030 if the barriers persist.

Policy Recommendations

To ameliorate the barriers to contraceptive use and accelerate progress of sub-Saharan African countries towards achievement of health for all by the year 2030, we proffer these policy recommendations:

1. Stakeholders of health in sub-Africa including health ministries, international health organisations such as the world health organisation and health-based NGOs should carry out intensified public education targeted at young people in order increase knowledge on contraceptives and reduce myths and misconceptions.
2. There is the need for community engagements to improve prohibitive social norms to make them more receptive towards contraceptive use and discussions regarding contraception
3. Contraceptive service provission should be made friendly. There should for instance, be a re-conscientisation of health professionals to improve their attitudes towards young people in the provision of contraceptive services to them. Privacy and confidentiality should be afforded the young people in their utilisation of services.
4. Efforts should be made by respective Sub-Sahara African countries to make public health facilities accessible for persons living with disabilities.
5. To reduce financial barriers, voucher schemes could be implemented for young people to access subsidised or free contraceptive services.

Strengths and limitations

Our study is the first effort at exploring the barriers and motivations for contraception among young people using purely qualitative studies. The fact that we reviewed only qualitative studies, however, meant that other relevant papers reporting barriers and motivations for contraceptive use among young people were expunged. It is, however, noteworthy that such studies could have

contributed to a better understanding of the totality of the barriers and motivators for young people's contraceptive use.

Abbreviations

FGD: Focus Group Discussion; LARC: Long-Acting Reversible Contraceptives; PRISMA: Preferred Reporting Items for Systematic Reviews and Meta-Analyses; STI: Sexually Transmitted Infection; SDG: Sustainable Development Goal; SSA: Sub-Sararan Africa; WHO: World Health Organisation

Declarations

Ethical approval:

Not applicable

Consent for publication

Not applicable

Availability of data and materials

All the data used for our review are contained within the manuscript

Competing interests

None declared

Funding

None

References

1. Sedgh G, Singh S, Hussain R. Intended and Unintended Pregnancies Worldwide in 2012 and Recent Trends. Stud FamPlann. 2014 Sep;45(3):301–14.
2. Unsafe abortion: global and regional estimates of the incidence of unsafe abortion and associated mortality in 2008. 6th ed. Geneva: World Health Organization; 2011. Available from: whqlibdoc.who.int/publications/2011/9789241501118_eng.pdf[cited 2016, Nov 10].
3. Vlassoff M, Singh S, Onda T. The cost of post-abortion care in developing countries: a comparative analysis of four studies. Health Policy Plan. 2016 Oct;31(8):1020–30.
4. Contraception and Unintended Pregnancy in Uganda [Internet]. Guttmacher Institute. 2016. Available from: https://www.guttmacher.org/fact-sheet/contraception-and-unintended-pregnancy-uganda.
5. Kweku M, Amu H, Awolu A, Adjuik M, Ayanore MA, et al. Community-Based Health Planning and Services Plus programme in Ghana: A qualitative study with stakeholders in two Systems Learning Districts on improving the implementation of primary health care. PloS One. 2020;15(1):e0226808.

6. Amu H, Nyarko SH. Preparedness of health care professionals in preventing maternal mortality at a public health facility in Ghana: a qualitative study. BMC health services research. 2016;16(1):252.
7. World Health Organisation. Adolescent health; 2020. https://www.who.int/southeastasia/health-topics/adolescent-health
8. Diedrich JT, Klein DA, Peipert JF. Long-acting reversible contraception in adolescents: a systematic review and meta-analysis. Am J Obstet Gynecol. 2017 Apr;216(4):364.e1-364.e12.
9. Bellizzi S, Palestra F, Pichierri G. Adolescent women with unintended pregnancy in low- and middle-income countries: reasons for discontinuation of contraception. J PediatrAdolesc Gynecol. 2019 Nov 9;
10. Luchters S, Bosire W, Feng A, Richter ML, King'ola N, Ampt F, et al. "A Baby Was an Added Burden": Predictors and Consequences of Unintended Pregnancies for Female Sex Workers in Mombasa, Kenya: A Mixed-Methods Study. PLOS ONE. 2016 Sep 30;11(9):e0162871.
11. Kebede A, Abaya SG, Merdassa E, Bekuma TT. Factors affecting demand for modern contraceptives among currently married reproductive age women in rural Kebeles of NunuKumba district, Oromia, Ethiopia. ContraceptReprod Med. 2019;4:21.
12. Dasa TT, Kassie TW, Roba AA, Wakwoya EB, Kelel HU. Factors associated with long-acting family planning service utilization in Ethiopia: a systematic review and meta-analysis. ContraceptReprod Med. 2019;4:14.

13. Engelbert Bain L, Zweekhorst MBM, Amoakoh-Coleman M, Muftugil-Yalcin S, Omolade AI-O, Becquet R, et al. To keep or not to keep? Decision making in adolescent pregnancies in Jamestown, Ghana. PLoS ONE. 2019;14(9):e0221789.
14. Weldegebreal R, Melaku YA, Alemayehu M, Gebrehiwot TG. Unintended pregnancy among female sex workers in Mekelle city, northern Ethiopia: a cross-sectional study. BMC Public Health. 2015 Jan 31;15:40.
15. Habte D, Teklu S, Melese T, Magafu MGMD. Correlates of Unintended Pregnancy in Ethiopia: Results From a National Survey. PLOS ONE. 2013 Dec 9;8(12):e82987.
16. Hamdela B, G/mariam A, Tilahun T. Unwanted Pregnancy and Associated Factors among Pregnant Married Women in Hosanna Town, Southern Ethiopia. PLOS ONE. 2012 Jun 22;7(6):e39074.
17. The Joanna Briggs Institute. Joanna Briggs Institute Reviewers' Manual: 2014 edition. Adelaide: The Joanna Briggs Institute, 2014
18. Uman LS. Systematic reviews and meta-analyses. Journal of the Canadian Academy of Child and Adolescent Psychiatry. 2011; 20(1):57.
19. Patient DR, Orr NM. What teenagers and young adults have to say about condoms and using condoms. AIDS analysis Africa. 2000;10(6):11.
20. Otoide VO, Oronsaye F, Okonofua FE. Why Nigerian adolescents seek abortion rather than contraception: evidence from focus-group discussions. International family planning perspectives. 2001:77-81.
21. Castle S. Factors influencing young Malians' reluctance to use hormonal contraceptives. Studies in family planning. 2003;34(3):186-99.

22. Flaherty A, Kipp W, Mehangye I. ‘We want someone with a face of welcome’: Ugandan adolescents articulate their family planning needs and priorities. Tropical doctor. 2005 Jan;35(1):4-7.
23. Tabane NS, Peu MD. Perceptions of female teenagers in the Tshwane District on the use of contraceptives in South Africa. curationis. 2015;38(2):1-7.
24. Ochako R, Mbondo M, Aloo S, Kaimenyi S, Thompson R, Temmerman M, Kays M. Barriers to modern contraceptive methods uptake among young women in Kenya: a qualitative study. BMC public health. 2015;15(1):118.
25. Hall K, Manu A, Morhe E, Zochowski M, Boakye A, Challa S, Loll D, Dozier J, Harris L, Adanu R, Dalton VK. Understanding “Bad Girl:” qualitative findings on stigma and adolescent sexual and reproductive health in Ghana. Contraception. 2015;92(4):393-4.
26. Hokororo A, Kihunrwa AF, Kalluvya S, Changalucha J, Fitzgerald DW, Downs JA. Barriers to access reproductive health care for pregnant adolescent girls: a qualitative study in Tanzania. Actapaediatrica. 2015 Dec;104(12):1291-7.
27. Capurchande R, Coene G, Schockaert I, Macia M, Meulemans H. “It is challenging… oh, nobody likes it!”: a qualitative study exploring Mozambican adolescents and young adults’ experiences with contraception. BMC women's health. 2016 Dec;16(1):48.
28. Hall KS, Manu A, Morhe E, Challa S, Loll D, Dozier J, Zochowski M, Boakye A, Harris L, Adanu R, Dalton V. Stigma and the social environment of adolescent sexual and reproductive health in ghana: understanding “bad girl”. Journal of Adolescent Health. 2016 Feb 1;58(2):S4.

29. Burke E, Kébé F, Flink I, van Reeuwijk M, le May A. A qualitative study to explore the barriers and enablers for young people with disabilities to access sexual and reproductive health services in Senegal. Reproductive health matters. 2017 Jun 12;25(50):43-54.
30. Hall KS, Manu A, Morhe E, Dalton VK, Challa S, Loll D, et al. Bad girl and unmet family planning need among Sub-Saharan African adolescents: the role of sexual and reproductive health stigma. Qualitative research in medicine & healthcare. 2018 May 30;2(1):55.
31. Rokicki S, Merten S. The context of emergency contraception use among young unmarried women in Accra, Ghana: a qualitative study. Reproductive health. 2018 Dec;15(1):1-0.
32. Haider TL, Sharma M. Barriers to family planning and contraception uptake in sub-Saharan Africa: a systematic review. International quarterly of community health education. 2013;33(4):403-13.
33. Radovich E, Dennis ML, Wong KL, Ali M, Lynch CA, Cleland J, Owolabi O, Lyons-Amos M, Benova L. Who meets the contraceptive needs of young women in sub-Saharan Africa?. Journal of Adolescent Health. 2018;62(3):273-80.
34. Frost JJ, Lindberg LD, Finer LB. Young adults' contraceptive knowledge, norms and attitudes: associations with risk of unintended pregnancy. Perspectives on Sexual and Reproductive Health. 2012 Jun;44(2):107-16.
35. Nguyen HN, Liamputtong P, Murphy G. Knowledge of contraceptives and sexually transmitted diseases and contraceptive practices amongst young people in Ho Chi Minh City, Vietnam. Health care for women international. 2006 Jun 1;27(5):399-417.
36. Craig AD, Dehlendorf C, Borrero S, Harper CC, Rocca CH. Exploring young adults' contraceptive knowledge and attitudes: disparities by race/ethnicity and age. Women's Health Issues. 2014;24(3):e281-9.

37. Kenny B, Hoban E, Pors P, Williams J. A qualitative exploration of the sexual and reproductive health knowledge of adolescent mothers from indigenous populations in Ratanak Kiri Province, Cambodia. Rural and remote health. 2019;19(4):5240.
38. Ahanonu EL. Attitudes of healthcare providers towards providing contraceptives for unmarried adolescents in Ibadan, Nigeria. Journal of family & reproductive health. 2014;8(1):33.
39. Tilahun M, Mengistie B, Egata G, Reda AA. Health workers' attitudes toward sexual and reproductive health services for unmarried adolescents in Ethiopia. Reproductive Health. 2012;9(1):19.
40. Yakubu I, Salisu WJ. Determinants of adolescent pregnancy in sub-Saharan Africa: a systematic review. Reproductive health. 2018;15(1):15.

Table 1: Background of studies included in the final review

**Author(s)**	**Year**	**Country**	**Contraceptives of focus**	**Age groups**	**Data collection methods**	**Sample size**
Patient et al. [19]	2000	South Africa	Condoms	15-24	FGD	88
Otoide et al. [20]	2001	Nigeria	All	15-24	FDG	149
Castle [21]	2003	Mali	All	15-24	IDI	84

Flaherty et al. [22]	2005	Uganda	All	14-20	FGD and general Discussions	29 in FGD and >500 in general discussions
Tabane&Peu [23]	2015	South Africa	All	15-19	IDI	15
Ochako et al. [24]	2015	Kenya	Modern Contraceptives	15-24	IDI	34
Hall et al. [25]	2015	Ghana	All	15-24	IDI	63
Hokororo et al. [26]	2015	Tanzania	All	15-20	FGD	49
Capurchande et al. [27]	2016	Mozambique	All	15-24	IDI, FGD, informal conversation, and observation	42
Hall et al. [28]	2016	Ghana	All	15-24	IDI	63
Burke et al. [29]	2017	Senegal	All	18-24 (with disabilities)	IDI and FGD	144
Hall et al. [30]	2018	Ghana	All	15-24	IDI	63
Rokicki&Merten [31]	2018	Ghana	Contraceptive pills	18-24	IDI	32

Table 2: Motivations for contraceptive use among young people in sub-Saharan Africa

**Main theme**	**Sub-theme**	**Studies**
Social support	From friends and peers	Tabane&Peu[23]<br>Burke et al. [29]
	From family	Hall et al. [30]<br>Burke et al. [29]
	Others	Tabane&Peu[23]
Protection of identity	Anonymity (of purchase points[vending machines])	Patient & Orr [19]<br>Burke et al. [29]
	Confidentiality (associated with patent medicine stores)	Otoide et al. [20]<br>Burke et al. [29]
Ready availability, affordability, and access	Accessibility	Patient & Orr [19]<br>Burke et al. [29]
	Availability (of condoms)	Castle [21]
	Affordability (low cost of condoms)	Castle [21]
Effectiveness in preventing unintended pregnancy and STIs	Preventing unintended pregnancy	Castle [21]<br>Tabane & Peu[23]<br>Rokiciki & Merten (2018)
	Preventing STIs	Castle [21]
Others	The need to maximise fertility in future (so as to gain status through child bearing)	Castle [21]
	Service providers being of the same sex with the young people	Flaherty et al. [22]
	Perceived quality of Pharmacy shops	Burke et al. [29]

Table 3: Barriers to contraceptive use among young people in sub-Saharan Africa

Main theme	Sub-theme	Codes	Studies
Personal	Myths and misconceptions	Lack of trust in contraceptives (especially condoms)	Patient & Orr [19]<br>Rokicki & Merten[31]
		Colour and size of available condoms considered unsuitable	Patient & Orr [19]
		Contraceptive use encourages promiscuity and straying (cheating)	Ochako et al. [24]<br>Burke et al. [29]<br>Rokicki&Merten[31]
		Contraceptive use reduces sexual pleasure	Ochako et al. [24]<br>Capurchande et al. [27]<br>Rokicki & Merten[31]
		There is a risk of future infertility with contraceptive use	Otoide et al. [20]<br>Castle [21]<br>Flaherty et al. [22]<br>Ochako et al. [24]
		Perceived ineffectiveness of contraceptives in preventing conception	Otoide et al. [20]<br>Hokororo et al. [26]<br>Capurchande et al. [27]<br>Rokicki&Merten[31]
	Lack/Inadequate knowledge	Poor knowledge on the mechanism of action of contraceptives and on how to utilise them	Otoide et al. [20]<br>Flaherty et al. [22]<br>Tabane & Peu[23]<br>Hokororo et al. [26]<br>Capurchande et al. [27]<br>Rokicki & Merten [31]
		Lack of reliable, trusted and non-judgemental sources of information on contraceptives	Flaherty et al. [22]<br>Burke et al. [29]<br>Rokicki&Merten[31]
	Negative attitude towards contraceptive use	Lack of personal motivation and willingness to utilise contraception	Tabane & Peu [23]
		Contraceptive use (mainly condom) is boring, stressful, too much of a responsibility, and clinical	Patient & Orr [19]<br>Tabane & Peu [23]<br>Capurchande et al. [27]
	Known side effects of contraceptives	Weight gain, headache, bleeding, high blood pressure, and disruption of menstrual cycle	Otoide et al. [20]<br>Tabane&Peu[23]<br>Ochako et al. [24]

			Capurchande et al. [27]<br>Rokicki&Merten[31]
	Financial challenges	Unaffordability of contraceptives and contraceptive services	Burke et al. [29]<br>Rokicki&Merten[31]
Societal based	Social consequences of contraceptive use	Divorce	Castle [21]
		Accusations of witchcraft	Castle [21]
		Stigma	Hall et al. [25]<br>Hokororo et al. [26]<br>Capurchande et al. [27]<br>Hall et al. [28]
		Tag of being promiscuous	Hall et al. [30]
	Social norms	Disproval of contraceptive use by friends and colleagues	Tabane&Peu[23]
		Disproval of contraceptive use by family and larger society	Tabane&Peu[23]<br>Burke et al. [29]<br>Hall et al. [30]
		Societal prohibition of discussions on issues concerning contraception	Capurchande et al. [27]<br>Burke et al. [29]<br>Rokicki&Merten[31]
		Contraception being considered an issue only for females	Capurchande et al. [27]
		Religious prohibitions	Burke et al. [29]
Health systems-based	Lack of privacy and confidentiality at health facilities		Flaherty et al. [22]<br>Hokororo et al. [26]<br>Burke et al. [29]
	Negative attitude of health professionals	Being treated disrespectfully	Flaherty et al. [22]
		Being entirely refused contraceptive services	Flaherty et al. [22]<br>Hokororo et al. [26]
		Being ignored teaching about contraceptives	Tabane&Peu[23]
		Discrimination	Burke et al. [29]<br>Hall et al. [30]
	Long waiting time		Hokororo et al. [26]
	Poor communication between health professionals and young people	Overly technical language used at health facilities	Capurchande et al. [27]<br>Burke et al. [29]
		Power asymmetry in communication between health professionals and young people	Capurchande et al. [27]
	Physical inaccessibility of	Staircases are unfriendly having to be accompanied by someone to	Burke et al. [29]

	health facilities	facilitate access	

Click here to access/download;Figure;Prismaflowchart.docx

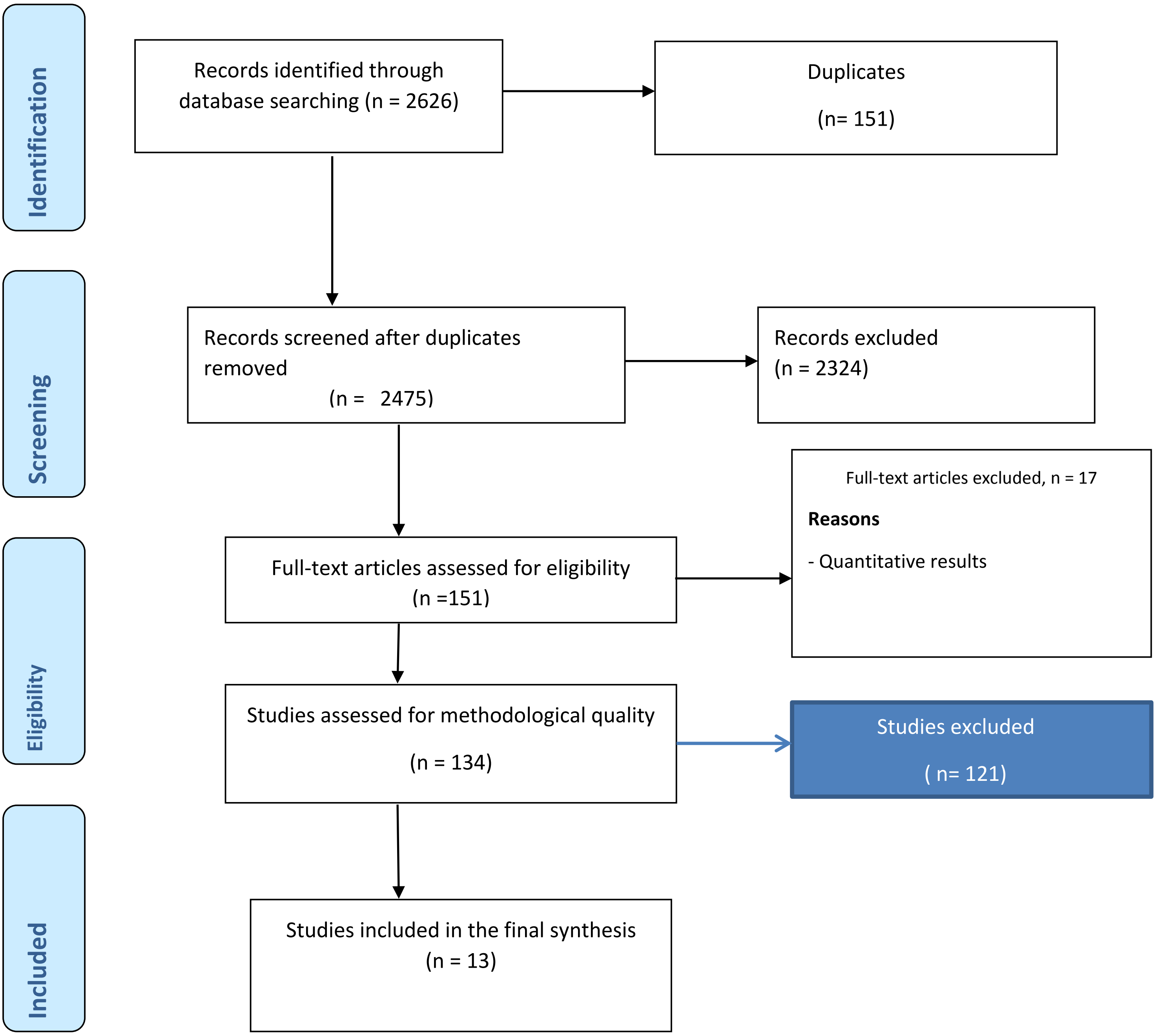

Figure 1: Multistep process for study selection and inclusion

Click here to access/download
Supporting Information
PRISMA Luchuo checklist (5).doc
