# Supplementary material for: Barriers and motivators of contraceptive use among young people in Sub-Saharan Africa: A systematic review of qualitative studies
Source: PLoS One. 2021 Jun 4;16(6):e0252745. doi: 10.1371/journal.pone.0252745 (PMC8177623; doi:10.1371/journal.pone.0252745)
Supplement: Attachment — Submitted filename: Response to Reviwers CommentsA-Reviewplosone.doc [file pone.0252745.s004.doc]

**Response to Reviewers**

At this time, please address the following queries:

a. Please clarify the sources of funding (financial or material support) for your study. List the grants or organizations that supported your study, including funding received from your institution.

**Response: This study did not receive any funding.**

b. State what role the funders took in the study. If the funders had no role in your study, please state: “The funders had no role in study design, data collection and analysis, decision to publish, or preparation of the manuscript.”

**Response: No funding was received for this study**

c. If any authors received a salary from any of your funders, please state which authors and which funders.

**Response: No salaries were received from funders.**

d. If you did not receive any funding for this study, please state: “The authors received no specific funding for this work.”

**Response:** “The authors received no specific funding for this work.”

Please include your amended statements within your cover letter; we will change the online submission form on your behalf.

**Response:**

4.We note that you have indicated that data from this study are available upon request. PLOS only allows data to be available upon request if there are legal or ethical restrictions on sharing data publicly. For information on unacceptable data access restrictions, please see [http://journals.plos.org/plosone/s/data-availability#loc-unacceptable-data-access-restrictions](http://journals.plos.org/plosone/s/data-availability#loc-unacceptable-data-access-restrictions).

**Response:**

In your revised cover letter, please address the following prompts:

a) If there are ethical or legal restrictions on sharing a de-identified data set, please explain them in detail (e.g., data contain potentially identifying or sensitive patient information) and who has imposed them (e.g., an ethics committee). Please also provide contact information for a data access committee, ethics committee, or other institutional body to which data requests may be sent.

**Response:**

5. Review Comments to the Author

Reviewer #1:

Authors need to go through the PDF document of the manuscript where reviewer comments are included.

**Response: this has been done and all issues raised by the reviewer have been pasted in the “response to reviewers” document and duly addressed**

The objective/question of the review was on three areas but only two of the questions were answered with the review. One question remains to be answered.

1. Search was conducted from a small number of data bases were searched.

Response: Thank you for this comment. Based on previous studies including those published in Plos One, we believe that the number of databases searched were exhaustive enough to ensure we retrieve all relevant publications. We actually searched PubMed, EMBASE, Ebsco/PsycINFO and Scopus. Searching other sources may only create further duplication of papers as these are the most popular databases available.

2. Too many papers (4457) were retrieved from which only 13 were eligible. Maybe the search terms used were not refined enough?

Response: Our study focused only on qualitative studies which was the reason for the rather small number of papers included in the final review. We have also detailed the search tersm in the methods section and believe that the terms used were exhaustive enough to produce the expected results.

3. The review has not answered one of the questions, Prevalence and usage.

**Response: The focus of this systematic review was only on the barriers and motivations for contraceptive uptake among young people.**

4. The protocol mentioned Prevalence and usage as one of the questions to be answered.

**Response: The main review has focused only on the barriers and motivations**

5. Enough number of data bases were not searched from.

Response: Thank you for this comment. Based on previous studies including those published in Plos One, we believe that the number of databases searched were exhaustive enough to ensure we retrieve all relevant publications. We actually searched PubMed, EMBASE, Ebsco/PsycINFO and Scopus. Searching other sources may only create further duplication of papers as these are the most popular databases available.

6. This maybe one of the reasons why you had too many non-eligible studies during the search. Restriction of time of study and/or publication is one thing to seriously consider.

Response: We agree with the reviewer that this is a plausible explanation of the high number of non – eligible studies we had. However, empirical qualitative research on the subject was not very rampant, and we wanted to be an inclusive as possible.

7. What about during a situation of failure to reach at consensus between the two reviewers? You need to have a third tie breaker reviewer in the team.

**Response: Even though a third reviewer was on standby, no such disagreements emerged between the two reviewers which required his inputs. That is why it has not been mentioned in the manuscript.**

8. Prevalence and usage?

**Response: This has not been explored in our current review even though contained in the registered protocol.**

9. Physical inaccessibility of buildings by the persons with disability" should be discussed here as one of the major barriers.

**Response: This has been done. See page 11.**

10. "Physical inaccessibility of buildings by the persons with disability" should be part of the conclusion section as a major barrier.

**Response: This has been done. See page 12.**

11. It is very difficult to reach at this type of conclusion based on this systematic review.

**Response: We have refined the statement to indicate that some countries, especially the ones from which the studies were reviewed, may not be able to achieve the SDG target with the persistence of the barriers identified. See page 12.**

Reviewer #2:

This is a systematic review on Barriers to and motivations for contraceptive use among adolescents and young women in Sub-Saharan Africa. Since low contraceptive usage is a challenge African countries are grabbling with, synthesising knowledge to identify gaps is important for public health.

Introduction: Pg3 The age range for young people includes adolescents. So why separate adolescents and young people in your population of interest?

**Response: We have revised the manuscript to focus only on young people.**

Methods: Pg 4 "the reviewers met" does not connote an objective process. Why was it not done independently and if necessary a third person adjudicates in the event of indecision?

**Response: the phrased has been revised accordingly (See page 5). Also, while a third reviewer was actually on standby, no serious disagreements in terms of what to include in the final review emerged and so that reviewer did not intervene.**

Policy recommendations: There is no indication from your write up on what policies countries have in place with regards to contraceptive usage for readers to appreciate if your policy recommendations are necessary. As it is no one knows what the countries within the review are doing.

**Response: A writ-up has now been included in the discussion in this regard. See page 12.**

General comments:
1. There are so many recommendations authors could give. For instance taking a look at Table 1, the age groups of included studies indicate a gap. As indicated in the introduction the age group is 10-24 years but the selected studies lack on age group 10-13 who are also sexually active.

**Response: A recommendation has been provided in this regard. See page 14.**

2. One would have expected that as what is known is being synthesised authors will identify gaps that need closing with research to win the low contraceptive battle. Because, bringing together what is available in the articles is not enough. This is concluded like a research based on a primary data. Gaps must be identified. Therefore as it is, a major revision is needed.

3. There's the need for editing to correct typographical errors

**Response: This has been done. An English language expert has proof-read the manuscript.**

4. Authors failed to indicate why the focus is on qualitative studies.

**Response: This has been done. See page 5.**
