# Supplementary material for: Barriers and motivators of contraceptive use among young people in Sub-Saharan Africa: A systematic review of qualitative studies
Source: PLoS One. 2021 Jun 4;16(6):e0252745. doi: 10.1371/journal.pone.0252745 (PMC8177623; doi:10.1371/journal.pone.0252745)
Supplement: Attachment — Submitted filename: Reviewers second round comments.docx [file pone.0252745.s005.docx]

**Reviewer’s comments 2**

- **Title:** it is better if written as “Barriers and motivators of contraceptive use among young people in Sub Saharan Africa: a systematic review of qualitative studies”.
- **Key words:** replace “Motivation” with “Motivators”.
- **Methods:** you need to include in methods section that a third reviewer was standby to be involved if disagreement happens between the two reviewers but there was need for it.
- Some editorial work is still needed.
